# An Integrated Neuronal Model of Claustral Function in Timing the Synchrony Between Cortical Areas

**DOI:** 10.3389/fncir.2019.00003

**Published:** 2019-02-05

**Authors:** Trichur R. Vidyasagar, Ekaterina Levichkina

**Affiliations:** ^1^Department of Optometry and Vision Science, University of Melbourne, Parkville, VIC, Australia; ^2^Florey Institute of Neuroscience and Mental Health, Parkville, VIC, Australia; ^3^Australian Research Council Centre of Excellence in Integrative Brain Function, University of Melbourne Node, Melbourne, VIC, Australia; ^4^Institute for Information Transmission Problems, Russian Academy of Sciences, Moscow, Russia

**Keywords:** claustrum, neural synchrony, cross-frequency coupling, visual cortex, visual search, attention

## Abstract

It has been suggested that the function of the claustrum (CL) may be to orchestrate and integrate the activity of the different cortical areas that are involved in a particular function by boosting the synchronized oscillations that occur between these areas. We propose here a model of how this may be done, thanks to the unique synaptic morphology of the CL and its excitatory and inhibitory connections with most cortical areas. Using serial visual search as an example, we describe how the functional anatomy of the claustral connections can potentially execute the sequential activation of the representations of objects that are being processed serially. We also propose that cross-frequency coupling (CFC) between low frequency signals from CL and higher frequency oscillations in the cortical areas will be an efficient means of CL modulating neural activity across multiple brain regions in synchrony. This model is applicable to the wide range of functions one performs, from simple object recognition to reading and writing, listening to or performing music, etc.

## A Region That Integrates Brain Activity

For purposeful and useful interaction with the external world, the brain needs to integrate information processed in different parts of the nervous system, so that it can efficiently process sensory inputs, often from more than one modality, stored memories, emotional aspects of the situation, and executive and motor programmes needed for the chosen response. This requires the operation of many brain areas communicating with each other. Crick and Koch (2005) published a stimulating idea that in the claustrum (CL), the brain may have a central integrator essential for our unified sense of cognition and cohesive behavior. This insight was inspired by the anatomical connectivity between the CL and other brain regions and the synaptic organization within the nucleus itself. The CL connects reciprocally with almost every cortical area (Pearson et al., 1982; Tanné-Gariépy et al., 2002; Druga, 2014; Torgerson et al., 2015; Wang et al., 2017). Furthermore, CL has been found to be the most densely interconnected structure in the human brain (Torgerson et al., 2015), and its internal structure can facilitate rapid development of synchronized activity within adjacent activated regions of the CL (Crick and Koch, 2005; Smythies et al., 2014a; Kim et al., 2016). Crick and Koch (2005) suggested that the dendro-dendritic synapses in the CL, which could potentially include gap junctions (Shepherd and Greer, 1998), can rapidly transfer signals arriving from different cortical areas. However, in the only study done in awake behaving macaques specifically aiming to record from multimodal neurones that would support an integrating function for single claustral cells, Remedios et al. (2010) found mainly unimodal sensory cells responding either to visual or auditory stimuli but not to both. Recent rodent studies of claustral circuitry have also shown only very weak connections between principal claustrocortical neurons (Kim et al., 2016). Smythies et al. (2012, 2014a,b) considering a few different hypotheses about how the CL may nevertheless be involved in integrating the activity across many parts of the brain, suggested that the most likely way the CL could exert its integrative function may not be by convergence of signals from various cortical areas on to single claustral cells, but rather by aiding cortical areas to amplify the synchrony between themselves. Saalmann et al. (2012) showed that the pulvinar does a comparable function in the maintenance of a working memory trace in a spatially cued object identification task. They demonstrated that the memory of the object location was maintained by a local cluster of pulvinar cells, as observed in the high degree of local spike-field coherence in the 8–15 Hz range and leading to almost zero-lag synchrony between visual areas V4 and TEO. This finding was supported by Zhou et al. (2016), who found a similar result prior to the appearance of the stimulus array in their paradigm. These synchronized oscillations were related to the maintenance of a memory trace that would be needed in the immediate future. Could the CL be doing something similar with regard to the actual processing and integration of sensory information and the behavioral response?

## Claustrum Could Enhance Synchronized Neural Oscillations Between Cortical Areas

A common principle of the mammalian brain that is being recognized as a fundamental mechanism driving its perception, cognition and behavior is the existence of periodic oscillations of neural activity amongst groups of active cells (e.g., Engel et al., 1991; Buzsáki et al., 2013; Buzsáki and Schomburg, 2015). Such rhythmic coordination in excitability is ubiquitous in the brain, but varying in its power, phase and frequency between brain regions and also between tasks. Almost every cortical activity involving processing of sensory information, memory, executive prerogatives and/or behavioral output inevitably engages multiple cortical areas communicating with each other and providing feedforward, feedback or modulatory signals. A plausible mechanism for such inter-areal communication is “communication through coherence” (Bastos et al., 2015), where rhythmic synchronization in one group of neurons leads to modulations in the input gain at synapses that they make on a second group of neurones. Such communication through coherence has been well documented by a number of studies through simultaneous recordings from two different cortical areas in awake macaques performing visual attention tasks (Buschman and Miller, 2007, 2009; Saalmann et al., 2007; Gregoriou et al., 2009).

Smythies et al. (2014a,b) suggested that when two cortical areas that are mutually connected and in a particular task begin to synchronize their activities, their connections to the CL first lead to rapid development of intraclaustral synchrony. These claustral regions are then believed to cause an increase in the synchrony between the two cortical regions through their efferents back to the respective cortical targets. While this proposition addresses the lack of multimodal neurones in the CL and yet ascribes to the CL a central integrative function, it opens up a number of new questions. Most importantly: (1) What is the relationship between the claustrocortical and cortico-cortical synchronies, in particular, do they occur at the same frequency? (2) What is the trigger for getting the CL involved and what terminates the synchrony generated in the cortex by the CL, without letting it evolve into a reverberating or even epileptiform discharge?

## Our Hypothesis of “Punctuated Neural Synchrony”

**Figure 1:**
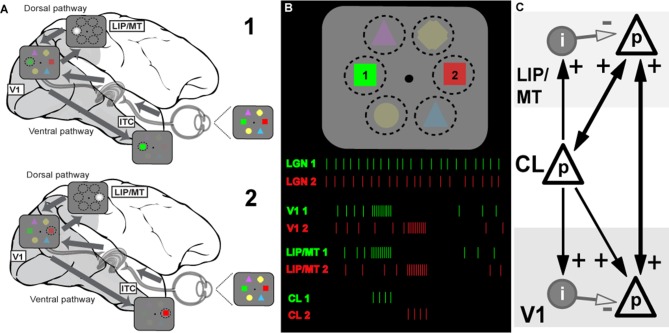
Model of the information flow during visual search and the role of claustrum (CL) in orchestrating this process. **(A)** Schematic depiction of the signal processing occurring in dorsal and ventral visual streams during visual search. Visual stimulus array is shown at the right side of the panel (and in B). Due to differences in speed of transmission, visual information first reaches areas of the dorsal stream (lateral intraparietal area/middle temporal, LIP/MT) *via* the faster magnocellular pathway. Dorsal stream areas provide spatial feedback to primary visual cortex (V1) and ventral stream areas in the form of a spotlight of attention (represented by bright gray circle). This feedback arrives at V1 by the time the slower parvocellular-mediated information reaches it, and facilitates further processing of stimulus just for the part of the visual scene where attention is directed to. The process serves to limit information overload in ventral stream areas of the inferotemporal cortex (ITC) by processing one item at a time, and helps to solve the binding problem as well (for more details see Vidyasagar, 1999). During visual search, parts of visual space containing salient features are processed sequentially, as represented by stages 1 and 2 corresponding to attentive processing of green and red figures of the visual array, respectively. **(B)** Visual search task and putative neuronal activities in key brain areas: lateral geniculate nucleus (LGN), V1, dorsal stream (LIP) and CL. The same visual stimulus array is presented at the top, spike responses are shown below as the attentional spotlight is focussed first on target 1 (green) and then on target 2 (red). The initial volley of excitatory burst from CL neurons to LIP/MT and to V1 is followed by feedforward inhibition which terminates the processing of each stimulus. Sustained activity of LGN provides relatively constant input for processing, the dorsal stream organizes attentional spotlights, and CL determines timing of item-by-item processing during visual search. **(C)** Claustral connections with V1 and dorsal stream areas (LIP/MT). p refers to excitatory cell in layer 4 of the cortex and i represents an inhibitory interneuron. The strength of functional connections is shown by the thickness of the arrows.

In this section, we outline a hypothesis for claustral function and illustrate it by applying it to “serial visual search”. Visual search is not only a very common function our brains perform, but is also a widely studied task in both humans and non-human primates. In most variations of this paradigm, one searches for a target among a number of items in a visual scene, with which the target shares one or more features. Early visual search experiments by Treisman and Gelade (1980) led them to propose a “feature integration theory” to explain how we detect objects in a cluttered visual scene and also how we are able to bind the attributes of each object before identification. This highly influential model proposes that a “spotlight of attention” selects at a time one particular object in the visual scene to be processed in detail and then moves on to others until the target is found. As a neural correlate of the feature integration model, it has been proposed (Vidyasagar, 1999; Bullier, 2001) that the dorsal cortical stream and its top-down feedback to the primary visual cortex (area V1) and to ventral stream structures serially select, from a priority map in the posterior parietal cortex, one particular location for a short time (Figure 1A). This is then processed in detail by the ventral areas that deal with object recognition. Despite the functional localization in the primate brain with different areas and neurones being specialized for different attributes such as color and shape, the simultaneous processing of the attributes of only one object at any one time leads to the binding of features of that object alone. In doing this, serial search proceeds at a rate of 20–45 ms/item, depending upon task demands (Wolfe et al., 1998; Wolfe and Horowitz, 2004). This translates into largely a beta and low gamma frequency range (22.2–50 Hz). The main neurophysiological support for this claim arises from a number of studies: (1) Buschman and Miller (2009, 2010) show that covert shifts of attention in a visual search task is correlated with the cyclical oscillation of top-down prefrontal modulation of parietal activity occurring in the low gamma range; (2) there is a wealth of evidence for the role of lateral intraparietal area (LIP) in directing top-down attention to specific objects (Bisley and Goldberg, 2003, 2010; Saalmann et al., 2007; Corbetta and Shulman, 2011; Meehan et al., 2017); (3) experiments in behaving macaques have shown that the top-down attentional feedback modulation of an early visual area, middle temporal (MT or area V5) by the parietal area, LIP is mediated by synchronized oscillations from LIP driving MT neurones at topographically corresponding locations, in the frequency range 25–45 Hz (Saalmann et al., 2007); and (4) though such cyclical modulation has not been directly demonstrated in the dorsal stream feedback to area V1, attentional and contextual modulation of V1 responses to visual inputs has long been amply demonstrated (Vidyasagar, 1998; Brefczynski and DeYoe, 1999; Ito and Gilbert, 1999; Gandhi et al., 1999; McAdams and Reid, 2005; Vidyasagar and Pigarev, 2007). Given the extensive neurophysiological evidence for synchronized neural oscillations in mediating interareal communication (Buschman and Miller, 2007; Saalmann et al., 2007, 2012; Gregoriou et al., 2009), it is not too speculative to suggest that the feedback to primary visual cortex is also likely to be mediated by such oscillations (Graboi and Lisman, 2003; Vidyasagar, 2013).

Extending the above argument, we propose that the CL’s comprehensive reciprocal connections with almost all cortical areas and their unique internal morphology help to magnify the synchrony between cortical areas and also provide a behaviorally useful sequence of activation across the surface of the corresponding cortical areas, such as what is needed in tasks such as serial visual search. Claustral anatomy and its connectivity are likely to accomplish the above requirements. In Figure 1C, we show a simplified canonical circuitry which is applicable to any two or more cortical areas that are functionally connected to the CL in any particular situation, but here shown for a visual task. Taking serial visual search as example, we show on the right claustral efferents projecting to principal (p) cells in both V1 and the dorsal stream (here, marked as LIP/MT).

Afferents to input layers in cortical areas not only synapse on to the excitatory stellate and pyramidal cells, but also to local inhibitory interneurons. Studied most intensively in the primary visual cortex (Creutzfeldt and Ito, 1968; Ferster and Lindström, 1983; LeVay, 1986), such an input leads to a powerful and long-lasting inhibition. Such strong feed forward inhibition (FFI) following on the heels of an excitatory input leads to aborting the excitatory response of the target cells after the initial volley (Bruno, 2011). While FFI has been shown to generate oscillations in a local network (Kremkow et al., 2010), FFI from one area to another, here from CL to V1, would serve another additional purpose, namely terminating the initial excitation.

Figure 1B shows how this may function in the case of serial visual search. In a typical visual search task, both engagement and disengagement from the items are essential and furthermore, they should occur sequentially, shifting from one item to another until the target is found, and all of this governed by task priorities. It is now believed (reviewed in Bisley and Goldberg, 2010) that area LIP has a continuously updated priority map that governs the allocation of top-down attentional signal. This priority map itself is updated from a number of inputs—especially task demands as dictated by prefrontal executive areas and saliency of the targets themselves (Ipata et al., 2009; Bisley and Goldberg, 2010). We propose that while the serial engagement of attention is determined simply by the pecking order in the priority map, the disengagement comes from the termination of the synchronized oscillations by the claustrocortical connections with areas that respond to the attributes of the object at the prioritized location. We suggest that such termination and thus the disengagement from the attended item is brought about by the inhibitory volley of the FFI circuit. Since such inhibition is long-lasting, it may also be the neural basis of “inhibition of return” (Wang and Klein, 2010), well-known in the visual search literature. Our proposed role of CL in facilitating top-down attentional modulation is consistent with results of recent experiments in rodents (Mathur, 2014; Goll et al., 2015; Atlan et al., 2018; White et al., 2018). Interestingly, CL not only receives selective top-down attentional influences from the cortex, for example from the anterior cingulate cortex (White et al., 2018), but it also plays a critical role in suppressing auditory distractors in a visual task (Atlan et al., 2018). Such a function is probably related to CL’s role in helping to distinguish between relevant and irrelevant items as in a typical search task.

**Figure 2:**
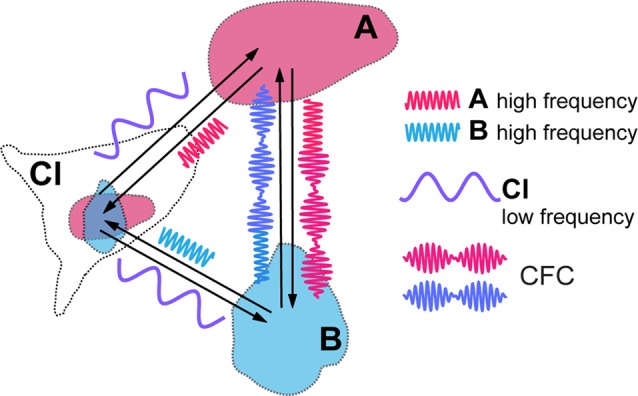
Cross-frequency coupling (CFC) of local high frequency activities generated in visual areas (A,B) caused by modulatory influence of claustral low frequency activity. Resulting amplitude modulation of synchronized high frequency activities of both areas frames the period during which signals are processed in synchrony between the topographically corresponding regions within the cortical areas, before they get aborted by the feed forward inhibition (FFI).

For attentive serial search to work in the fashion described above, we expect that any reciprocal connection from V1 to CL is weak or non-existent. As described earlier, serial search requires moving the spotlight of attention from one item of the scene to another until the target is found. Object recognition is known to occur largely in the ventral stream and it is believed to be facilitated by top-down modulation of incoming visual signals by feedback from the dorsal stream (Vidyasagar, 1999; Bullier, 2001). Once visual attention gets focussed on one object by the spotlight of attention, the CL may play little role in the more detailed processing by the ventral cortical areas. Finding the target would abort the FFI from the CL and the activity in V1 and the corresponding topographic locations in the various cortical areas would continue under focussed attention. Furthermore, if activity related to object locations are supposed to be “serially highlighted” for further processing by extra-striate areas such as LIP, MT, V4 and TEO for ultimate binding of the attributes of the object, such a scheme would be defeated if there are strong signals from every item to the CL, triggering reciprocal synchronizing volleys. In fact, many studies on the CL, while describing the widespread afferent connections from the CL to most association areas and the prefrontal cortex have emphasized the uncertainty of the projection from the primary sensory areas, including V1 in the primate (Druga, 2014; reviewed in Smythies et al., 2014a). There is also a cautionary note about the effectiveness of the V1 (area 17) to CL projection that has been described in the cat. LeVay and Sherk (1981), who studied connections between visual areas and CL in the cat, found that area 17 cells projecting to CL were just 3.5% of layer 6 cells and these were found predominantly in the peripheral rather than central visual field representation, whereas the claustral projections to area 17 were much heavier. Sherman and Guillery (2011) state that the layer 6 cells that project subcortically are class 2 glutamatergic cells that do not produce much spiking activity but only modulate responses mainly through metabotropic postsynaptic receptors. Thus, the claustral synchrony may get initiated and sustained, not so much by the sensory input to primary sensory areas, but rather by activity in higher areas such as LIP. Thereafter, as the enhanced synchrony between the representations of a particular object in different cortical areas (in Figure 2, V1 and LIP/MT) develops and then dies down with its termination by FFI, the next most salient location in LIP synchronizes with V1 and the corresponding locations in the CL also get activated and a new cycle of enhanced synchrony starts, to be in its turn terminated by the subsequent FFI.

Recent studies of the rodent CL have demonstrated the strong inhibitory influence that optogenetic stimulation of claustral outputs could have on cortical areas, namely on unit responses in the anterior cingulate cortex (White et al., 2018), the prefrontal cortex (Jackson et al., 2018; Narikiyo et al., 2018) and the auditory cortex (Atlan et al., 2018). We suggest that these inhibitory volleys represent the FFI needed to terminate activity in target areas as described above in our scheme. It is noteworthy that in all of these studies, the optogenetic excitation was effective in causing the inhibition in target cortical areas, but the initial excitatory response in the target area was rather weak (Narikiyo et al., 2018). This may be attributed to two factors: (i) the optogenetic stimulation does not resemble the usual synchronized oscillatory activity that may be needed to cause the excitatory oscillations as described above and in the section below on cross-frequency coupling (CFC) and (ii) the excitatory response would require temporal simultaneity of oscillatory inputs from other cortical areas.

Recent rodent studies have also elucidated a claustral circuitry that could be ideally suited to our proposed function of the CL (Kim et al., 2016, see their Figure 8), by possibly enabling another FFI circuit within the CL itself. While corticoclaustral inputs target individual claustrocortical (ClaC) cells monosynaptically and there are few direct connections among these principal, claustrocortical cells, the cortical inputs to CL provide strong stimulation to the parvalbumin (PV)-positive inhibitory interneurons, which are themselves strongly interconnected *via* both electrical and chemical synapses (Kim et al., 2016). This leads to a situation where synchronous activation signals from two different cortical areas to their reciprocally connected ClaC cells would set off a neural synchrony between the cortical areas and the CL, soon to be followed by an inhibitory volley that suppresses the claustral outputs.

Finally, when the target in a visual search task is found, the termination of all activity in the CL and the search itself may be brought about by stimulation of the kappa opioid receptors, the mRNA for which is particularly plentiful in the CL (Mansour et al., 1994). The high density of these receptors on claustral cells is a striking finding that needs particular consideration in any model of claustral function. The possible role of this receptor system in the larger integrative functions has been pointed out (Stiefel et al., 2014), since such receptor stimulation inhibits the release of GABA (Hjelmstad and Fields, 2003; Li et al., 2012) which in turn would disrupt the generation of oscillations within the CL and the claustral amplification of the synchrony between cortical areas. Activation of the kappa receptors inhibits both glutamate and GABA transmission (Hjelmstad and Fields, 2003), thus practically stopping excitatory activity as well as disrupting oscillations. We believe that a match between an object brought under the roving spotlight of attention and the representation of the expected object may abort the visual search through its effect on claustral kappa opioid receptors. While the kappa opioid system may be generally known for its dysphoric effects, particularly in producing the aversive and depressive effects in the case of drug abuse (Lalanne et al., 2014), the evolutionary reason for the kappa receptors are not likely to be related to drug addiction. Natural opioids acting on mu opioid receptor (MOR) and kappa receptors are known to lead to opposing effects in rats performing a behavioral task, the former to reinforcement of the related behavior and the latter to its termination (Shippenberg and Herz, 1986). Though stimulation of kappa receptors in the ventral tegmental area may be related to motivational and hedonic aspects (Spanagel et al., 1992), similar stimulation in other areas may have effects depending upon the function of those respective areas. Thus, their primary role may be simply in aborting neural synchrony in local circuits through their action on GABEergic transmission, besides the inhibition of the excitatory activity itself. We propose that until the visual search is completed, there is little stimulation of the claustral kappa-opioid receptors, but a specific input to the CL on finding the target, possibly from the prefrontal regions which are heavily linked to the CL (Reser et al., 2014), may disrupt neural oscillations in the CL and consequently its amplification of synchrony in various cortical regions.

Our model of claustral control of visual search is one convenient example for what we believe to be a description of claustral function in general. We believe that the proposed role of CL in sequencing neuronal activity is not restricted to the visual modality, but in line with its widespread cortical connections, CL can potentially modulate activity in all sensory cortices, association areas and also motor areas. Thus, we hypothesize that the CL might be instrumental in not only in binding the activity of different cortical regions by enhancing their synchrony, but also organizing all cortex-mediated processes in a sequential manner, as for example in language comprehension, language production and in organizing complex motor programs.

## Claustral Modulation of Other Brain Areas Through Cross-Frequency Coupling

CFC is being recognized as an efficient means of communication between two cortical areas and it is likely to play a critical role in mediating working memory and in enabling learning (Canolty and Knight, 2010; Lisman and Jensen, 2013; Hyafil et al., 2015). Blood-oxygen-level dependent (BOLD) connectivity between areas is best predicted by low frequency oscillations that determine the amplitude of gamma frequencies (Wang et al., 2012). Thus, in the above example, in target cortical areas such as LIP, MT and V1, the amplitudes of a higher, such as high beta or gamma, frequency rhythm may be modulated by, and thus nested within, a lower frequency, for instance theta, alpha or low beta, at which claustral efferents send out their modulating signals to their targets (Figure 2). We expect that each cycle of the low frequency signal from CL would allow sufficient number of high frequency cycles at its target areas to synchronize before the excitatory volley gets aborted by the FFI. While electrical stimulation of lateral geniculate nucleus (LGN) leads to disynaptically mediated inhibitory post-synaptic potentials in stellate cells in layer 4 of the primary visual cortex within a few milliseconds (Creutzfeldt and Ito, 1968; Ferster and Lindström, 1983), with visual stimulation the inhibition seen in intracellular recordings from the cat striate cortex develops gradually over many tens of milliseconds (Pei et al., 1994; Volgushev et al., 1995: Ringach et al., 1997). Both with such visual stimulation and with electrical stimulation (Viswanathan et al., 2011), the inhibition can however last many hundreds of milliseconds. Strong FFI caused by CL stimulation and mediated *in vivo* by relatively slow neuropeptide Y interneurons was also described in the prefrontal areas of rodents by Jackson et al. (2018), with the excitation/inhibition ratio of cortical pyramidal cells equalling just 0.25. Though one is yet to see similar studies done in the case of the primate CL, the window of opportunity for neural synchrony between relevant cortical regions to be amplified by claustral output is likely to be defined by the time course of the FFI circuit. It is possible that this time course may also be modulated by task demands and the state of vigilance.

The cyclical facilitation of processing of incoming visual signals in V1 would mean that sensitivity to visual stimuli could show periodic fluctuation, as indeed they do (Busch et al., 2009; Mathewson et al., 2009; VanRullen and Dubois, 2011). CFC with nested frequencies may also be critical for processing of stimuli at multiple temporal rates, such as graphemes/phonemes, and syllables and words during reading and speech perception (Graboi and Lisman, 2003; Vidyasagar, 2013). Through CFC, claustral output at one low frequency (delta, theta, alpha, or low beta) can modulate a range of oscillation frequencies (high beta or gamma) at cortical areas that are connected to each other in a task such as reading or visual search. Figure 2 is a simplified diagram of how this might function in the case of CL boosting synchrony between LIP/MT and V1. At this stage, it is too premature to speculate at what frequency the claustral assembly oscillates. It may be either always at the same frequency which is determined by its own morphology and resonance frequency or dictated by the area that triggers the synchrony in the first place or even under an executive command from the prefrontal cortex.

## Outstanding Questions for Future Studies

The model leads to a number of testable predictions. The following are some of the main questions for study.

1. In tasks such as visual search, the model predicts neural activity in CL driving synchronized activities in relevant cortical regions.
2. Claustral influence on cortical regions would exhibit two stages: an initial excitatory oscillation followed by strong inhibition.
3. The time course of the FFI from CL on cortical areas needs to be ascertained to test whether it permits synchrony between cortical areas.
4. Is the low frequency volley from CL mediating CFC fixed or is it dynamically modified by task demands?
5. In a visual search task, is there a roving wave of synchrony across the CL as the animal performs a search task, as the corresponding topographic locations in the CL serially facilitate the scan of the spotlight of attention?
6. Is there a rapid termination of intraclaustral synchrony and stimulation of GABAergic neurons as soon as the target is found?

Some of these questions need to be addressed in awake non-human primates. So far, with rare exceptions (Remedios et al., 2010, 2014) the primate CL has defied functional studies, due to its shape and anatomical location, but it is possible that with newer emerging techniques, the experiments are feasible.

## Conclusion

Our hypothesis suggests the existence of a functional circuit by which CL could play a vital role in communication between cortical areas by enhancing both the synchrony between cortical areas as well the amplitude of oscillations. The scheme has the advantage that though the connections between cortical areas themselves may not be structurally and functionally strong to develop enough synchrony, the boost given by the CL can help them to attain a degree of synchrony that will be functionally useful. Critical to this function is the unique claustral morphology (Kim et al., 2016) and the FFI circuit both within the CL and in its cortical targets, which are features considered to be characteristic of a system designed to amplify correlated neuronal activity (Bruno, 2011; Hu et al., 2014). The metaphor that Crick and Koch (2005) thought of, that the CL is like the conductor of an orchestra, is apt in more ways than one. In short, the punctuated synchrony we propose is akin to the conductor of an orchestra co-ordinating and inspiring a harmonious and smoothly punctuated symphony. In short, it is a *conductor of the synchrony* between cortical areas.

## Author Contributions

TV was responsible for the basic idea proposed in the article and drafting the first version. He also conducted some of the experiments that underpin crucial elements of the proposed theory. EL assisted in developing the basic idea to fit into a diagrammatic scheme and provided critical input towards the article’s intellectual content. EL also enhanced comprehension of the article with revisions and the illustrations.

## Conflict of Interest Statement

The authors declare that the research was conducted in the absence of any commercial or financial relationships that could be construed as a potential conflict of interest.
